# Mechanical Tuning of the Cell Microenvironment Using a Biomimetic Hydrogel System for Articular Cartilage Tissue Engineering

**DOI:** 10.1155/term/9947868

**Published:** 2026-05-31

**Authors:** Marloes van Mourik, Janne Spierings, Pinar Koca, Florencia Abinzano, Gabriele Addario, Corrinus C. van Donkelaar, Keita Ito, Jasper Foolen

**Affiliations:** ^1^ Orthopaedic Biomechanics, Department of Biomedical Engineering, Eindhoven University of Technology, Eindhoven, the Netherlands, tue.nl; ^2^ Institute of Interfacial Process Engineering and Plasma Technology IGVP, University of Stuttgart, Stuttgart, Germany, uni-stuttgart.de

## Abstract

Providing a functional pericellular matrix (PCM) by fine‐tuning the microenvironment of the articular chondrocytes (ACs) can greatly improve the outcomes of articular cartilage tissue engineering. While harvesting ACs with their PCM (chondrons) results in a low cell yield and a heterogeneous mixture of ACs and chondrons, microscale hydrogels could be used for mechanical tuning of the cell microenvironment. This may enable the use of stiffer bulk materials, improving the load‐bearing capacity of the construct. This study investigates the effect of microenvironmental stiffness, independent of total construct stiffness, on the regenerative performance of ACs (ECM and PCM synthesis). Additionally, we explored articular cartilage–derived progenitor cells (ACPCs) as a possible alternative to ACs in the presented system. ACs were cultured in a soft or stiff bulk hydrogel (GelMA) or were encapsulated in soft microgels and seeded into the stiff GelMA. Constructs were seeded in an ex vivo porcine chondral defect model and cultured for 28 days with dynamic mechanical stimulation using a compression‐sliding bioreactor. PCM and ECM quality were assessed through cell content analysis, immunofluorescent staining, histology, and measurements of GAG and collagen content. Cell encapsulation influenced ECM synthesis and PCM amount and completeness throughout the construct. Although the nonencapsulated groups showed stronger overall alcian blue staining, the encapsulated groups demonstrated more uniform matrix deposition throughout the depth of the tissue. Furthermore, ACPCs performed similarly to ACs. These findings suggest that the approach to differentially tune encapsulating and bulk hydrogel properties holds potential for future articular cartilage tissue engineering, and that ACPCs could be used as an alternative cell source.

## 1. Introduction

Articular hyaline cartilage is essential for the functioning of load‐bearing joints as it provides lubrication and absorbs mechanical loads [1, 2]. However, damage to the tissue can lead to degeneration and eventually osteoarthritis due to the tissue’s poor innate healing capacity [3]. The high prevalence of these chondral defects [4] and possibly debilitating outcomes make the restoration of damaged articular cartilage a pressing matter. Tissue engineering strategies have been developed to repair the tissue and restore biomechanical function [5–7]. Unfortunately, their capacity to provide a long‐lasting solution is limited as the regenerated tissues rarely remain load‐bearing [8, 9].

Articular chondrocytes (ACs) are highly mechanosensitive cells, altering their phenotypical behavior based on changes in environmental mechanical properties and loading [10]. The transduction of mechanical stimuli is largely determined by the properties of its microenvironment. In articular cartilage, chondrocytes are surrounded by a highly specialized pericellular matrix (PCM), together referred to as the “chondron.” While the extracellular matrix (ECM) is mainly comprised of Type‐II collagen and sulfated glycosaminoglycans (sGAGs) [11], the PCM contains some unique components like Type‐VI collagen and perlecan [12, 13]. The PCM has a pivotal mechanobiological role on ACs during joint loading, caused by the large difference between PCM (∼40 kPa) and ECM (up to 2 MPa close to the calcified region) stiffness [14, 15]. This causes stress shielding in high‐strain areas and strain amplification where the local strains are small [2, 14, 16, 17]. These properties are hypothesized to create a near‐uniform microenvironment for the cells throughout the depth of the tissue. The use of enzymatically isolated chondrons is beneficial for articular cartilage tissue engineering [18, 19] but yields low cell numbers and results in a mixture of chondrocytes and chondrons [20, 21]. As chondrons cannot be expanded [18], their clinical use requires a mixture with other cells [22, 23]. Finding an alternative method to facilitate ACs with an adequate microenvironment could advance the outcomes of cartilage repair strategies.

Current literature has shown that ACs thrive in hydrogels with a stiffness similar to the native PCM [24]. Studies using gelatin methacrylate (GelMA) [25, 26], agarose [27], and alginate [28] have shown that ACs produce proper cartilage‐like matrix in these conditions. However, when implanted in a load‐bearing joint, these materials are prone to failure since the mechanical demands of the joint are not met by these soft hydrogels [29]. Combining a soft hydrogel‐based cell microenvironment with a stiff bulk material could thus hold great potential, and the use of such an artificial microenvironment could be a good alternative to using chondrons. In an earlier study, we showed that ACs display chondrogenic behavior when encapsulated and cultured in soft microscale agarose hydrogels for up to 10 days [30]. A study by Fredrikson et al. [31] recently showed that human ACs produced a compact ring of Type‐VI collagen in 10 days in alginate microgels of a similar scale. Additionally, the expression of chondrogenic proteins was retained after the soft microgels were embedded in a stiff bulk hydrogel and mechanically loaded. However, this study did not analyze ECM synthesis, and the constructs were cultured for only 15 min. Studying local ECM and PCM production after a longer culture time will provide more insight into the cell behavior in such a system.

In addition to the lack of a PCM, the use of ACs for cartilage tissue engineering has other challenges. A high cell density is preferred to stimulate cartilaginous matrix deposition. Due to the low cell content of native articular cartilage, the use of autologous ACs requires *in vitro* expansion, which induces the loss of the chondrogenic phenotype [8, 32]. As an alternative to ACs, the use of articular cartilage–derived progenitor cells (ACPCs) has been suggested [33, 34]. Unlike ACs, ACPCs can maintain their chondrogenic phenotype after extensive passaging [35, 36] and can have superior ECM production when stimulated correctly [37]. Additionally, ACPCs are known to produce a PCM in soft agarose microgels similar to ACs [30], showing the potential of these cells as an alternative to ACs.

This study aims to investigate the effect of microenvironmental mechanical stiffnesses on local ECM and PCM synthesis by ACs, by lowering only the microenvironmental stiffness, independent of the total construct stiffness. In analogy to the native articular cartilage, we hypothesize that combining a soft microenvironment with a stiff bulk construct will stimulate superior synthesis of PCM and ECM. The soft microenvironment is required for the synthesis of chondrogenic matrix components. The stiff bulk material will allow mechanical stimulation to penetrate into lower areas of the construct, resulting in more uniform matrix production by the embedded cells. In testing this hypothesis, the compatibility of ACPCs was also investigated and compared to encapsulated ACs (Encap ACs), where the two cell types were expected to exhibit similar behavior based on earlier reports. If so, ACPCs would be a realistic alternative to ACs for cartilage tissue engineering purposes.

## 2. Materials and Methods

### 2.1. Experimental Setup

**FIGURE 1:**
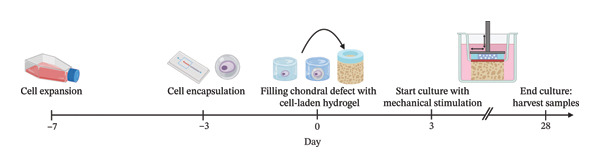
Schematic overview of the timeline used in this research.

To investigate the effect of different microenvironmental mechanical stiffnesses, a biomimetic approach was used to simulate the PCM and ECM stiffness. Cells were expanded *in vitro*, and four different experimental groups were created: (1) Low‐stiffness GelMA with ACs (LS GelMA, *n* = 6); (2) high‐stiffness GelMA with ACs (HS GelMA, *n* = 6); (3) HS GelMA with Encap ACs (*n* = 4); and (4) HS GelMA with encapsulated ACPCs (Encap ACPC, *n* = 6). Cells were encapsulated in agarose microgels using droplet‐based microfluidics to mimic the soft PCM. Encap ACPCs were precultured for 3 days to stimulate chondrogenic differentiation. Encap ACs were also precultured to have the same culture time for both cell types. The different combinations of cells and hydrogels were used in a porcine chondral defect model and cultured for 28 days in a compression‐sliding bioreactor to simulate the effect of mechanical loading on matrix synthesis (Figure 1). After 28 days, the PCM and ECM synthesis was analyzed by measuring cell content, immunofluorescent stainings, and histology, GAG and collagen content, and mechanical characterization of the constructs.

### 2.2. GelMA Synthesis and Characterization

Type B bovine bone gelatin (Gelita, Germany) was dissolved in MilliQ water at 40°C to prepare a 10% (w/v) gelatin solution. After adjusting the pH to 7.5 with 4 M NaOH, methacrylic anhydride (MAAnh) (Sigma‐Aldrich, Germany) was introduced to the gelatin solution at a controlled rate of 0.2 mL min^−1^ under continuous stirring. To achieve middle and high degrees of modification (DM), MAAnh was added in two different molar ratios: 1:1 and 1:2 to the free amino groups of gelatin, which were reported to be 0.35 mmol g^−1^ [38]. Following the complete addition of MAAnh, the reaction continued for 1 h. The reaction solution was then purified through cross‐flow filtration and subsequently frozen and lyophilized to obtain the final product.

The modification degrees were determined using ^1^H‐NMR spectroscopy. The lyophilized GelMA was dissolved in deuterium oxide (Deutero, Germany), and spectra were acquired using a Bruker Avance 500 spectrometer. The quantification of methacrylation degrees for low‐ and high‐DM GelMA batches was performed using the phenylalanine content procedure as described by Claaßen et al. [39], resulting in 0.170 ± 0.001 mmol/g (∼49%) and 0.334 ± 0.007 mmol/g (∼95%), respectively. The percentages are estimated based on a free amino group content of 0.35 mmol/g [38].

GelMA with a lower DM was used to produce LS GelMA, while a higher DM was used to produce HS GelMA. For both, 10% w/w GelMA containing 0.1% w/w Lithiumphenyl‐2,4,6‐trimethylbenzoylphosphinate (LAP, TCI, L0290) was prepared in DPBS (Sigma‐Aldrich, D8662) and cast into cylindrical molds. LS GelMA was directly photocrosslinked, while HS GelMA underwent a precooling step before photocrosslinking.

Unconfined compression tests were performed after the hydrogels reached their swollen equilibrium state, using a ZwickRoell Z005 (Zwick, Germany) equipped with a custom‐built measuring chamber at 37°C in DPBS. Cylindrical samples (Ø 8 mm) were compressed at a constant rate of 0.1 mm/s, without preload. The elastic modulus of acellular, fully swollen hydrogels was calculated from the linear region (0%–8% strain) of the stress–strain curve, using a custom Python script optimized for preload‐free data processing. Values of 26.82 kPa (±2.56 kPa; *n* = 6) and 410.77 kPa (±18.18 kPa; *n* = 6) were obtained for the LS GelMA and HS GelMA, respectively.

### 2.3. Articular Cartilage Harvest, Cell Isolation, and Cell Culture

Articular cartilage from healthy (without obvious macroscopic damage) bovine metacarpophalangeal joints was harvested and enzymatically digested according to a previously described protocol [20]. In short, fresh bovine metacarpophalangeal joints (*n* = 6 for ACs; *n* = 6 for ACPCs) obtained from the slaughterhouse were dissected and articular cartilage tissue was digested overnight with 0.15% collagenase Type‐II (17101‐015, Gibco, Thermo Fisher Scientific, Landsmeer, the Netherlands) and 0.1% hyaluronidase (H3506, Sigma‐Aldrich, Zwijndrecht, the Netherlands). After the enzymatic digestion, ACs were either frozen directly in freezing medium containing 90% fetal bovine serum (FBS, BCBV7611, Sigma‐Aldrich) and 10% dimethyl sulfoxide (DMSO, 276855, Sigma‐Aldrich) at −150°C or ACPCs were isolated from the obtained cells.

To extract the ACPCs from the total cell population, differential fibronectin adhesion was used, as described previously [30, 37]. In short, the cell suspension was added to a fibronectin‐coated culture flask, where ACPCs were allowed to adhere for 20 min at 37°C. Nonadherent cells were removed and discarded, and the remaining adherent cells were cultured up to Passage 2 in ACPC expansion media (Table 1) containing Dulbecco’s modified Eagle medium (DMEM) with GlutaMax (31966, Gibco), 10% FBS, 1% penicillin/streptomycin (P/S, m15070039, Thermo Fisher Scientific), 50 μg/mL L‐ascorbic acid‐2‐phosphate (AsAP, A8960, Sigma‐Aldrich), 1% MEM nonessential amino acids (NEAA, 11140050, Gibco), and 5 ng/mL recombinant human basic fibroblast growth factor (bFGF, 100‐18B, PeproTech, London, United Kingdom). After passage 2, ACPCs were frozen in freezing medium at −150°C until further use.

**TABLE 1:** All different medium compositions used in this study.

Medium supplements	AC expansion medium	ACPC expansion medium	AC droplet medium	ACPC droplet medium	Cartilage‐specific medium	Bone‐specific medium
FBS	10%	10%				10%
ITS + premix			1%	1%	1%	
P/S	1%	1%			1%	1%
Fungin					25 μg/mL	50 μg/mL
HEPES			1%	1%		
AsAP	50 μg/mL	50 μg/mL	1%	15	50 μg/mL	50 μg/mL
NEAA	1%	1%				
β‐glycerophosphate						10 mM
L‐proline					40 μg/mL	
bFGF		5 ng/mL				
TGF‐ β1			10 ng/mL		10 ng/mL	
Dexamethasone			0.1 μM			
BMP‐9				100 ng/mL		

*Note:* The base medium used is DMEM with GlutaMax (Gibco 31966).

Before use, ACs and ACPCs were thawed and expanded before encapsulation in the hydrogels. ACs were seeded at 2000 cells/cm^2^ and grown in expansion media (Table 1) until Passage 1. ACPCs were seeded at 2000 cells/cm^2^ and grown in expansion media (Table 1) with 5 ng/mL bFGF, to stimulate proliferation and chondrogenic potential [40], until Passage 3. Both cell types were harvested after 5–7 days of culture at ca. 80% confluence. Cells were released from the culture plastic using 0.25% trypsin–ethylenediaminetetraacetic (EDTA) phenol red (25200, Thermo Fisher Scientific) and collected for further use.

### 2.4. Cell Encapsulation

**FIGURE 2:**
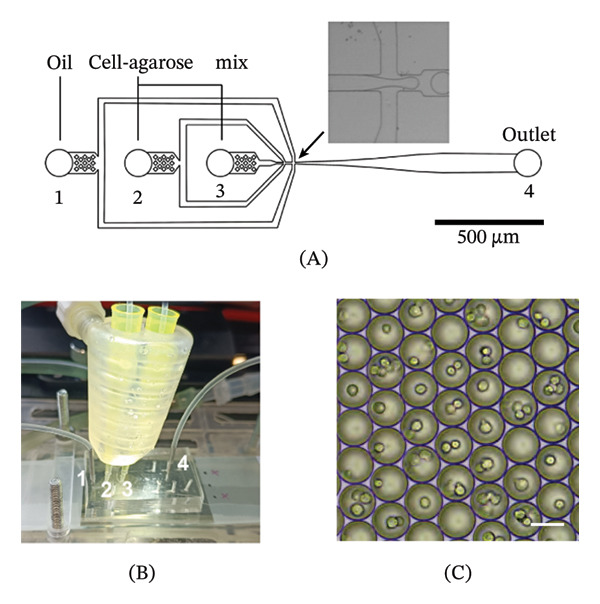
Overview of the microfluidic setup and cell‐laden agarose microgels. Schematic overview of the microfluidic chip design used for droplet‐based microgel formation (A). The intersection between the aqueous and oil phases, where the emulsion is formed is indicated with the arrow. Overview of the microfluidics setup showing the microfluidic chip and the pipette tips, containing the cell‐agarose solution, inserted in the heating device (B). The numbers correspond to the inlets and outlets indicated in A. Agarose droplets containing ACs in emulsion, directly after droplet formation (C). Scale bar = 50 μm. Adapted from van Mourik et al. [30].

ACs and ACPCs were encapsulated in soft microgels using a previously described method [30]. In short, a previously developed 3‐inlet polydimethylsiloxane (PDMS) microfluidic device [41] (Figure 2A) with a custom‐made heating device [42] (Figure 2B) was used to encapsulate the cells using droplet‐based microfluidics. Both cell types were suspended at 20 ∗ 10^6^ cells/mL in 1% w/v ultra‐low‐gelling‐temperature agarose (A5030, Sigma‐Aldrich) in phosphate‐buffered saline without calcium and magnesium (DPBS, 14190250, Thermo Fisher Scientific). The equilibrium modulus of the agarose is ∼10 kPa [43].

During droplet formation, the cell‐agarose solution and continuous phase, containing HFE‐7500 fluorinated oil (Novec 7500), were set to 5 μL/min and 40 μL/min, respectively. During droplet formation, a sample of droplet suspension was collected and imaged with an EVOS microscope (Thermo Fisher Scientific, Figure 2C). After collection, the agarose was allowed to gel for 15 min at 4°C and the emulsion was broken using 20% 1H,1H,2H,2H‐perfluoro‐1‐octanol (PFO, 370533, Sigma‐Aldrich) in HFE‐7500. The cell‐laden microgels were precultured for 3 days in 6‐well plates with cell culture inserts (0.4 μm pore size, Sterlitech, Auburn, Washington, United States) containing DMEM (31966), 1% ITS + premix (354352, Corning, Life Technologies Europe, Bleiswijk, the Netherlands), 1% HEPES (15630‐080, Gibco), and 1% AsAP. Additionally, ACs were stimulated with 10 ng/mL transforming growth factor‐beta 1 (TGF‐β1, 100‐21, PeproTech) and 0.1 μM dexamethasone (50‐02‐2, Merck Life Sciences NV, Amsterdam, the Netherlands) and ACPCs were stimulated with 100 ng/mL bone morphogenetic protein‐9 (BMP‐9, 120‐07, PeproTech) for 3 days (Table 1).

### 2.5. Osteochondral Explant Harvest and Cell‐Laden Hydrogel Preparation

Osteochondral explants were harvested from the femoral condyles of porcine knees, obtained from a local abattoir, using a dental coring drill (Ø 10 mm, MF‐dental). During the drilling procedure, sterile cooled PBS, supplemented with 2% P/S, was used to limit the effect of the drilling‐induced heat. The osteochondral plugs were stored in bone‐specific medium consisting of high‐glucose DMEM (Gibco 31966) supplemented with 10% FBS, 1% P/S, 50 μg/mL fungin (InvivoGen, Toulouse, France), 50 μg/mL L‐ascorbic acid‐2‐phosphate (Merck), and 10 mM β‐glycerophosphate (Sigma‐Aldrich) at 37°C until the defect was created.

Using a flat‐bottomed end‐mill and a custom‐made centering guide, a full‐depth cartilage defect (Ø 6 mm) was created in the center of the explants. Afterward, the defects were filled with cell‐laden hydrogels. In more detail, ACs were embedded in 10% (w/v) low‐ or high‐DM GelMA at 5 ∗ 10^6^ cells/mL. To make the hydrogel precursor solutions, 10% (w/v) low‐ or high‐DM GelMA was dissolved in LAP solution. For the Encap ACs or ACPCs, 10% high‐DM GelMA was mixed with cell‐laden microgels at a 4:1 ratio, resulting in approximately 4 ∗ 10^6^ cells/mL. The cartilage defect was filled with the cell‐laden hydrogel precursor solution and covered with a glass coverslip. The HS GelMA, Encap AC, and Encap ACPC samples were then precooled at 4°C for 1 h to allow physical crosslinking to increase the stiffness of the GelMA [44]. Afterward, the samples were UV‐crosslinked for 10 min at room temperature with a wavelength of 365 nm at a 7‐cm distance and source intensity of 0.58–1.49 mW/cm^2^ (VL‐4.LC, A. Hartenstein GmbH [Würzburg, Germany]). LS GelMA was UV‐crosslinked without precooling.

### 2.6. Explant Culture With Mechanical Stimulation

Osteochondral defect explants with cell‐laden hydrogels were cultured for 28 days at 37°C, 5% O_2_, and 5% CO_2_ in a previously developed double‐chamber compression‐sliding bioreactor culture platform [45]. The upper compartment was filled with cartilage‐specific medium (DMEM [31966]) supplemented with 1% ITS + premix, 1% P/S, 25 μg/mL fungin, 40 μg/mL L‐proline (Merck), 50 μg/mL AsAP, and 10 ng/mL TGF‐β1 and the lower compartment with bone‐specific medium (Table 1). The culture media were refreshed every 3–4 days.

**FIGURE 3:**
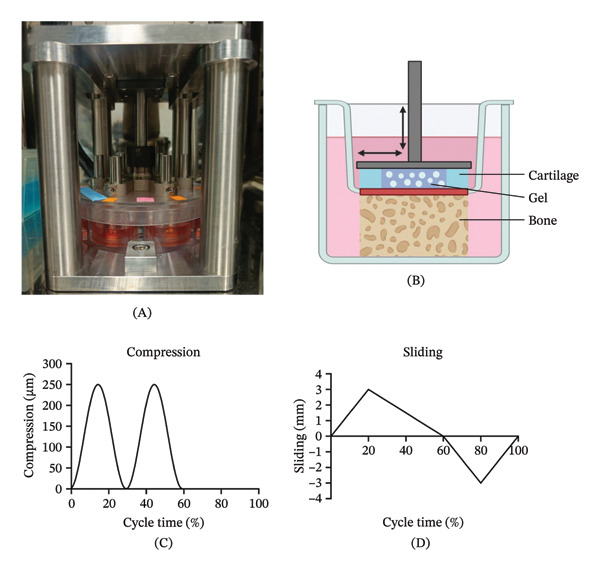
Picture of the bioreactor in the incubator showing the culture plate that can house six samples (A). Schematic overview of the culture chamber of the bioreactor showing the direction of the mechanical loading with arrows. (B). Compression and sliding pattern applied by the bioreactor to mimic human gait (C, D).

After 2 days of resting, the samples were subjected to a loading regime mimicking human gait [45] (250 μm compression, ±3 mm sliding, frequency 1 Hz, for 2 h/day, 5 days/week) in a compression‐sliding bioreactor (LifeTec Group BV, Eindhoven, the Netherlands; Figure 3). All samples were harvested after 28 days of culture.

### 2.7. Mechanical Testing

The mechanical properties of the created samples at Day 0 (LS GelMA, *n* = 6; HS GelMA, *n* = 6; Encap AC, *n* = 5; Encap ACPC, *n* = 6) were evaluated by performing an indentation test using a MicroTester (CellScale, Waterloo, Canada), with a 1.5‐mm spherical indenter connected to the end of a 0.56‐mm microbeam (cantilever). The osteochondral plugs with the cell‐laden hydrogel were immersed in PBS at room temperature during testing. To start, the microbeam was positioned just above the hydrogel samples with the bead centered on the defect. The samples were then indented for five cycles, in which the microbeam generated cyclic compression by applying a vertical indentation of 10% of the sample’s cartilage thickness, manually set per sample using the MicroTester’s camera and crosshairs. Each cycle consisted of a 20‐s loading phase, at a velocity of 0.5% indentation per second, and a 10‐s unloading phase, without rest in between. During the test, the machine records the indentation force and the indentation depth, which are calculated (known beam stiffness) and measured on the optically recorded deflection of the cantilever [46].

Afterward, to quantify the apparent modulus, the data were analyzed using an in‐house developed MATLAB algorithm (2021a, The MathWorks Inc., Natick, MA), using the Hertz contact model for a spherical indenter [47] in combination with the algorithm proposed by Huth et al. [48]. In short, a least squares fitting function was used to fit the Hertz model to the experimental force data. Starting from the contact point, the fitting range was incrementally increased, and the apparent modulus was calculated at different indentation depths. Using a standard deviation filter, the data subset that presented similar apparent moduli was found. Subsequently, within this data subset, the apparent modulus that corresponded to the fit with the least chi‐square was chosen as the representative apparent modulus. The Poisson’s ratio was assumed to be 0.44 [49].

### 2.8. Biochemical Assays

After mechanical testing, half of each sample was used for biochemical analysis (LS GelMA, *n* = 6; HS GelMA, *n* = 6; Encap AC, *n* = 5 for Day 0, *n* = 4 for Day 28; Encap ACPC, *n* = 6). The other half was used for histology (LS GelMA, *n* = 4; HS GelMA, *n* = 5; Encap AC, *n* = 3; Encap ACPC, *n* = 4). Using a scalpel, the gel was separated from the surrounding cartilage and bone, and the gel was stored at −80°C. All samples were freeze‐dried for 24 h and afterward digested in papain digestion buffer (5 mM L‐cysteine hydrochloride [Sigma‐Aldrich] plus 5 mM Na_2_EDTA [VWR], containing 130 μg/mL papain [Sigma‐Aldrich]) at 60°C overnight.

The total DNA content was quantified using the High Sensitivity dsDNA Qubit fit (Thermo Fischer Scientific). According to the manufacturer’s description, 190 μL Working Solution was mixed with 10 μL sample. The DNA content was calculated based on the value given by the fluorometer and normalized to the sample dry weight, obtained before sample digestion.

GAG content was quantified using a dimethylmethylene blue (DMMB) assay based on the protocol of Farndale et al. [50]. In short, digested samples were 1: 10 diluted in digestion buffer, and then, 40 μL of samples and standards (chondroitin sulfate from shark cartilage; Sigma‐Aldrich, C4348) was mixed with 150 μL DMMB solution [46 μM 1‐9‐dimethylene blue (Sigma‐Aldrich) plus 40.5 mM glycine (VWR) and 40.5 mM NaCl (Merck), pH 3.0]. The absorbance was measured at wavelengths 540 nm and 595 nm, and these values were subtracted from each other (540–595 nm). GAG concentrations were calculated based on a standard curve and normalized to sample dry weight obtained before digestion.

Hydroxyproline was quantified as a measure of collagen based on the protocol of Huszar et al. [51]. In short, digested samples were hydrolyzed with 16M NaOH solution (VWR) for 10 min at 120°C and neutralized using 1.4 M citric acid (Merck). The chromophore was formed via the addition of Chloramine‐T solution (Sigma‐Aldrich) and aldehyde/perchloric acid solution (150 mg 4‐[dimethylamino]benzaldehyde (Sigma‐Aldrich) per mL N‐propanol (VWR), 70% perchloric acid [Fluka], and 4% MilliQ solution). Absorbance was measured at a wavelength of 550 nm. The amount of hydroxyproline present in the samples was calculated based on standards (hydroxyproline [Sigma; H5534]) and normalized to sample dry weight, obtained before digestion.

### 2.9. Determination of Cell Death

As an indication of cell death, lactate dehydrogenase (LDH) was quantified in the culture media, sampled every time the culture media was replaced during the 4‐week culture period. The media samples from the first 2 weeks were diluted 1:1 in PBS. The samples from Weeks 3 and 4 were used without dilution. The LDH reaction mixture was prepared according to the manufacturer’s instructions (11644793001, Roche, Sigma‐Aldrich) and mixed with an equal amount of medium supernatant. Absorbance was measured at wavelength 490 nm immediately after the mixture and every 10 min to ensure values were within the standard curve (known NADH concentrations [10107735001, Roche, Sigma‐Aldrich]). The maximum LDH release was measured using samples that were frozen at −20°C to ensure maximum cell death.

### 2.10. Histology

Histological samples were fixed overnight at 4°C in zinc formalin fixative (Z2902, Sigma‐Aldrich). The bone part of the osteochondral plugs was decalcified using Immunocal formic acid bone decalcification solution (STL14141, StatLab, Fisher Scientific) for 48 h, refreshing the decalcification solution after 24 h. Samples were placed in a humidified chamber, and the hydrogels with the surrounding cartilage were kept moist with PBS. For cryopreservation, all samples underwent treatment to minimize frost damage, using sucrose (Fischer Scientific) in dH_2_O. Consequently, samples were incubated in 10% sucrose for 2 h, 15% sucrose for 2 h, 20% sucrose overnight, 30% sucrose for 8 h, and 1:1 30% sucrose with Tissue‐Tek O.C.T. Compound (OCT; Sakura Finetek) overnight, all at 4°C. Samples were kept in OCT at room temperature for 3–6 h before snap freezing using liquid nitrogen. The cryopreserved samples were kept at −80°C until further use.

Cryosections were cut at 5 μm thickness using a Leica CM1950 cryostat (Leica Biosystems, Amsterdam, the Netherlands) and collected on SuperFrost microscope slides (17214894, Epredia, Fisher Scientific). Cryosections were dried overnight and stored at −20°C until staining.

Cryosections were stained for sGAGs using Alcian Blue and nuclei using Mayer’s hematoxylin. Cryosections were defrosted for 10 min and rehydrated in dH_2_O for 5 min. Samples were stained in 1% Alcian Blue 8GX (A‐5268, Sigma‐Aldrich) solution for 30 min. Then, samples were washed and stained with Mayer’s hematoxylin (MHS16, Sigma‐Aldrich) for 10 min. After staining, the samples were dehydrated with 1 × 95% ethanol, 3 × 100% ethanol, and 2x xylene, all for 1 min. Samples were mounted with Entellan (1079610500, Merck) and covered with a glass cover slip. Sections were visualized using a brightfield microscope (20x, 0.55 NA, Dmi8, Leica Microsystems, Wetzlar, Germany).

### 2.11. Immunofluorescence

Cryosections were thawed for 10 min and rehydrated in PBS for 10 min. Samples were treated with a 0.1% Triton X‐100 (Merck) permeabilization solution and antigens were retrieved by incubating samples with 0.05% pepsin (P7000, Sigma‐Aldrich) in 10 mM HCl for 10 min at 37°C. Antigens were blocked with 2% normal goat serum and 4% bovine serum albumin (BSA) in PBS for 30 min at room temperature. Samples were stained with an antibody cocktail for either Type‐I collagen (1:100, ab138492, Abcam) and Type‐II collagen (1:50, II‐116B3, DSHB) or Type‐VI collagen (1:200, ab6588, Abcam) and perlecan (1:100, sc‐33707, Santa Cruz Biotechnologies, Dallas, Texas, United States). All primary antibodies were diluted in 10% blocking solution with PBS and incubated overnight at 4°C. Subsequently, samples were stained with secondary antibodies (Col‐I: 1:200, A21244; Col‐II: 1:200, A21127; Col‐VI: 1:200, A21244; Perlecan: 1:200, A11006; all from Thermo Fisher Scientific) and 4′,6‐diamidino‐2‐phenylindole (DAPI, 1:5000, D9542, Sigma‐Aldrich) for 1 h at room temperature. Stained sections were visualized using a fluorescence microscope (10x, 0.3 NA; 20x, Axio Observer 7, Zeiss, Oberkochen, Germany).

### 2.12. Microscopy Data Analysis

To study the distribution of the investigated matrix proteins and the structure of the synthesized PCM, microscopy data of the Alcian blue and immunofluorescence stainings were analyzed using CellProfiler (v 4.2.6, Broad Institute, Cambridge, Massachusetts, United States). For each sample, regions of interest (ROIs) were selected in the top, middle, and bottom regions of the hydrogel construct (Figure S1, Supporting Information).

Fluorescent microscopy images were segmented and structural parameters (cell density and PCM features) were determined as described previously [30]. In short, all images were converted to grayscale images, and an illumination correction was applied. The images were segmented using a three‐class Otsu thresholding method, which identified the objects for further analysis. The number of cell nuclei was determined based on the number of segmented DAPI objects per ROI.

The Alcian blue staining was evaluated by determining the total intensity of the ROI. The Alcian blue images were converted to grayscale using the unmix colors functionality of CellProfiler. The total image intensity was determined as the sum of all pixel intensity values. To correct for the cell content, the total intensity was normalized to the number of nuclei obtained from the fluorescent microscopy image analysis.

The structure of the PCM was evaluated by determining the PCM completeness (area and coverage). The PCM coverage was determined by calculating the overlap between a 2‐pixel region around the cell nuclei and the Type‐VI collagen and perlecan stainings, similar to our previous report [30].

### 2.13. Statistical Analysis

All statistical analyses were performed using GraphPad Prism Version 10 (GraphPad Software, Inc., San Diego, CA). Normality of the data was tested using a Shapiro–Wilk test. Parametric tests were used when the dataset was normally distributed; otherwise, a nonparametric test was used. To compare the experimental groups LS GelMA, HS GelMA, and Encap AC at a specific time point, a one‐way ANOVA or Kruskal–Wallis test with Tukey’s or Dunn’s multiple comparison tests, respectively, was performed. To compare different time points within an experimental group or Encap AC to Encap ACPC at the same time point, an independent *t*‐test or Mann–Whitney test was performed. For the LDH, to compare between the experimental groups LS GelMA, HS GelMA, and Encap AC at a specific time point, a one‐way ANOVA or Kruskal–Wallis test, with, respectively, Tukey’s or Dunn’s multiple comparison tests was performed. To compare different time points within an experimental group or Encap AC to Encap ACPC at the same time point, a Friedman or mixed‐effect analysis test with, respectively, Dunn’s or Tukey’s multiple comparison tests, or independent *t*‐test or Mann–Whitney test was performed. For the microscopy analysis, the ROIs within one experimental group were compared using a repeated‐measures one‐way ANOVA or Friedman’s test with, respectively, Tukey’s or Dunn’s multiple comparisons test. Significance was set at *p* < 0.05. Results are expressed as the mean ± standard deviation.

## 3. Results

### 3.1. Incorporating Encapsulated Cells Resulted in a Reduction of the Apparent Modulus of the Stiff Bulk Hydrogel

The mechanical properties of the constructs were assessed on Day 0 to verify the apparent modulus of the created constructs. HS GelMA, indeed, showed a higher apparent modulus than the LS GelMA group (*p* = 0.01, one‐way ANOVA; Figure S2, Supporting Information). The effect of embedding agarose microgels in HS GelMA on the bulk apparent modulus was nonsignificant (HS GelMA = 371 ± 148 kPa; Encap AC = 261 ± 43 kPa), but also not significantly higher than LS GelMA (LS GelMA = 151 ± 66 kPa; Supporting Figure 1).

### 3.2. GelMA Hydrogels Laden With (Encapsulated) Cells Were Successfully Cultured in an Ex Vivo Setup

**FIGURE 4:**
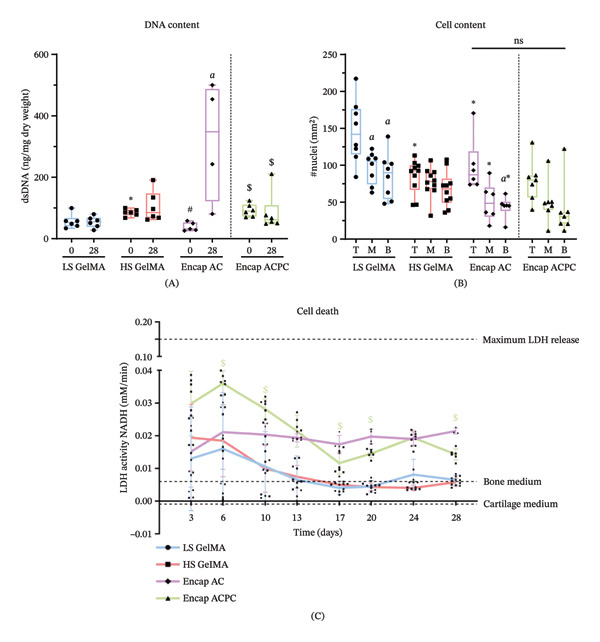
GelMA hydrogels laden with (encapsulated) cells preserved their viability. (A) The dsDNA content in all experimental groups. Between experimental groups, ^∗^indicates a significant difference from LS GelMA, # indicates a significant difference to HS GelMA, $ indicates a significant difference from Encap AC, and *a* indicates a significant difference between Day 0 and Day 28. (B) Depth‐dependent cell content based on the microscopy images on Day 28. Between experimental groups, ∗ indicates a significant difference to LS GelMA. Between regions, *a* indicates a significant difference to the top. (C) LDH activity measured in the cell culture supernatant. Between experimental groups, $ indicates a significant difference to Encap AC. To improve visibility, not all significant differences are indicated in figures. A complete overview of the exact *p*‐values can be found in Supporting Tables 1 and 2.

DNA quantification revealed that the number of cells was not significantly different between Day 0 and Day 28 samples within each group, indicating no cell proliferation, except for the Encap AC group (*p* = 0.01, independent *t*‐test), which also showed a large standard deviation (Figure 4A). On Day 0, HS GelMA showed a significantly higher dsDNA content than both LS GelMA (*p* = 0.03, one‐way ANOVA) and Encap AC (*p* = 0.002, one‐way ANOVA).

**FIGURE 5:**
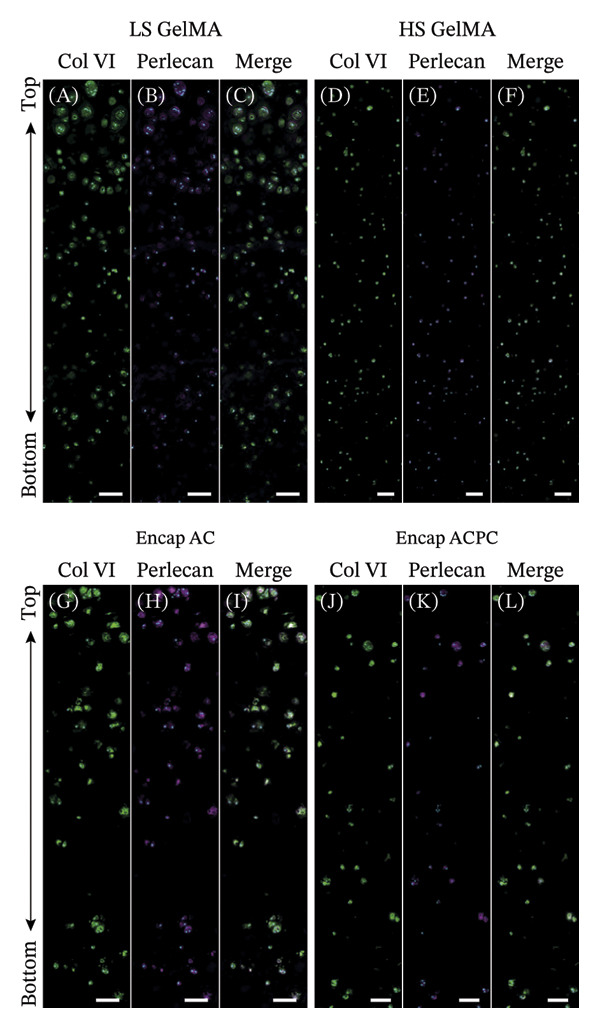
The staining of PCM components showed that ACs produce a superior PCM when a soft microenvironment is combined with a stiff bulk hydrogel. No evident differences between ACs and ACPCs can be observed. Immunofluorescent stainings of Type‐VI collagen (green; A, D, G, H), perlecan (magenta; B, E, H, K), and a combined projection (C, F, I, L) with cell nuclei (cyan) are depicted. Scale bar = 100 μm.

**FIGURE 6:**
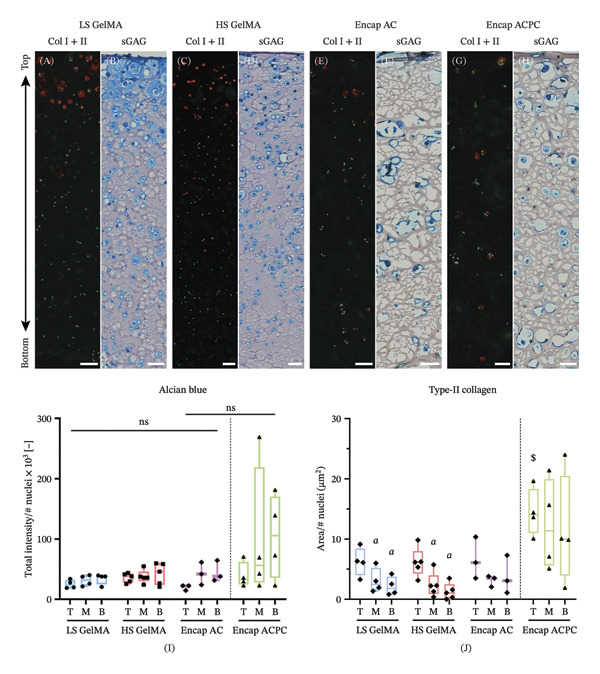
Staining of ECM components showed large variations in cell content and depth‐dependent chondrogenic matrix synthesis. Immunofluorescent stainings of Type‐I collagen (gray), Type‐II collagen (red), and cell nuclei (cyan) are depicted in A, C, E, and G. Alcian blue staining for sGAGs is depicted in B, D, F, and H. Scale bar = 100 μm. The relative intensity of alcian blue (I) and Type‐II collagen (J) staining, normalized to the number of cell nuclei in the ROI. Between experimental groups, ^∗^ indicates a significant difference to LS GelMA, # indicates a significant difference to HS GelMA, and $ indicates a significant difference to encapsulated ACs. Between regions, *a* indicates a significant difference to the top and *b* indicates a significant difference to the middle. A complete overview of the exact *p*‐values can be found in Supporting Table 1 and 2.

Microscopy images revealed differences in cell content between depth regions and experimental groups (Figures 5 and 6). Therefore, the number of cell nuclei/mm^2^ was determined based on the microscopy images. The number of cell nuclei/mm^2^ in the microscopy images of LS GelMA throughout the construct was significantly higher than Encap ACs (top: *p* = 0.046; middle: *p* = 0.002; bottom: *p* = 0.013). In the HS GelMA, only the cell content in the top region was significantly lower compared to similar regions in the LS GelMA (*p* = 0.003) (B). These results suggest more cell proliferation in the top region of the LS GelMA compared to the other groups.

Besides the differences in cell content between groups, significant differences were observed at different depths within experimental groups. Both LS GelMA and Encap ACs contained significantly more cells in the top region than in the middle (LS GelMA: *p* = 0.018; Encap AC: *p* = 0.039) and bottom regions (LS GelMA: *p* = 0.0045; Encap AC: *p* = 0.013). As the cell distribution throughout the samples was homogeneous on Day 0 (Figures S3, S4, Supporting Information), the cells in the top region proliferated more compared to the other areas. Such differences were not observed for the HS GelMA group (Figure 4B).

Next to that, LDH activity in the supernatant, as a measure of cell death, decreased throughout the culture in all groups except the Encap AC (Figure 4C). On Day 28, the measured LDH activity in the Encap AC group was significantly higher than that in the other groups, but still far from the maximum LDH release (positive control).

### 3.3. PCM Structure Is Significantly Improved by Cell Encapsulation

Cartilage‐like matrix synthesis was observed in all groups. Synthesis of PCM was observed in all groups, as all samples were positive for both Type‐VI collagen and perlecan (Figure 5). Differences in PCM components and structure were observed within samples and between groups.

**FIGURE 7:**
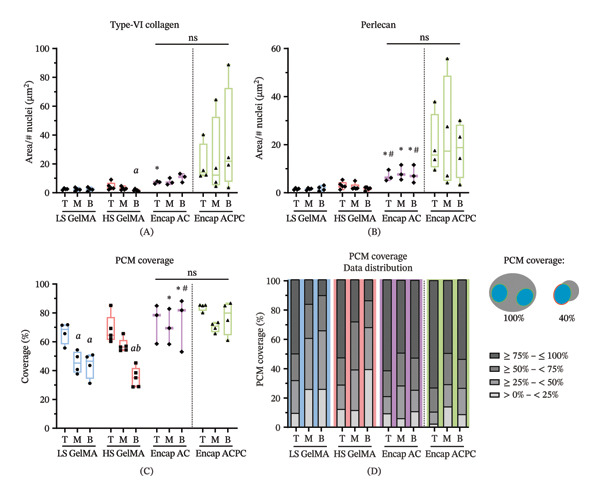
Encapsulation significantly improved the synthesis of perlecan and the overall PCM structure. Encapsulated ACs and ACPCs showed no significant differences in PCM synthesis. The relative area of Type‐VI collagen (A) and perlecan (B) staining, normalized to the number of cell nuclei in the analyzed ROI. PCM structure is expressed in PCM coverage around cell nuclei (C, D). Experimental groups are compared using a one‐way ANOVA. Between experimental groups, ^∗^ indicates a significant difference to LS GelMA, # indicates a significant difference to HS GelMA, and $ indicates a significant difference to encapsulated ACs. Between regions, *a* indicates a significant difference to the top and *b* indicates a significant difference to the middle. A complete overview of the exact *p*‐values can be found in Supporting Table 1 and 2.

The microscopy images show differences in PCM synthesis between the experimental groups. The relative area of perlecan staining, normalized to the number of cell nuclei, significantly increased after encapsulation compared to all regions of the LS GelMA (top: *p* = 0.0026 middle: *p* = 0.019; bottom: *p* = 0.0083) and the top (*p* = 0.015) and bottom (*p* = 0.0059) regions of the HS GelMA (Figure 7B). This indicates that the encapsulation of ACs drastically improved perlecan synthesis and PCM maturation throughout the construct. No significant differences were found between regions, except for the Type‐VI collagen content in the top (4.47 μm^2^ ± 2.75) and bottom (1.70 μm^2^ ± 0.622) of the HS GelMA (Figure 7A; *p* = 0.036).

The semiquantitative analysis of the microscopy data allowed for the evaluation of the coverage of the PCM formed during the 28‐day culture, which was assessed by measuring the percentage of the nuclei surrounded by a PCM (Figure 7C, D). This showed that the PCM completeness in the lower regions of the gels without encapsulation was inferior to the PCMs produced at the top. This was evident by the lack of PCM coverage (LS GelMA top vs middle: *p* = 0.0081; top vs bottom: *p* = 0.0054; HS GelMA top vs bottom: *p* = 0.0007; middle vs bottom: *p* = 0.0077) in the lower regions of the construct. Additionally, the distribution of the PCM coverage showed that in the lower regions, for both LS and HS GelMA, the number of cells with 75%–100% coverage decreased, while the number of cells with 0%–25% coverage increased (Figure 7D). This was confirmed visually by the lack of PCMs in the microscopy panels of the lower regions of the construct (Figure 5C,F,I,L).

With cell encapsulation, these depth‐dependent differences in PCM structure were significantly diminished, since no significant differences in PCM coverage were observed between the regions (Figure 7C). Additionally, the differences in PCM coverage data distribution were more similar throughout the regions for the Encap ACs. Compared to the other groups, the PCM coverage (LS GelMA vs Encap AC: middle: *p* = 0.0073; bottom: *p* = 0.015; HS GelMA vs Encap AC: bottom: *p* = 0.0027) significantly increased after encapsulation. These results indicate that the combination of the low‐stiffness microenvironment and high‐stiffness bulk hydrogel had a positive effect on PCM completeness throughout the constructs.

### 3.4. Encapsulation of ACs in Agarose Microgels Does Not Lead to an Overall Improvement of the Chondrogenic Effect

After observing an improved PCM with cell encapsulation, the effect on ECM synthesis was assessed. Thus, biochemical properties were evaluated. The hydroxyproline assay, as a measure of collagen [51], was in this setup unable to detect collagen production by the cells since the hydroxyproline content inherent to GelMA overshadowed the potential collagen production by the cells. Next to that, differences in GAG content between empty LS and HS GelMA were observed, likely caused by background noise. It was, therefore, decided to normalize the data by subtracting the mean GAG content at Day 0 from the measured concentrations at Day 28. This allows for a more accurate comparison of GAG production between the different experimental groups.

**FIGURE 8:**
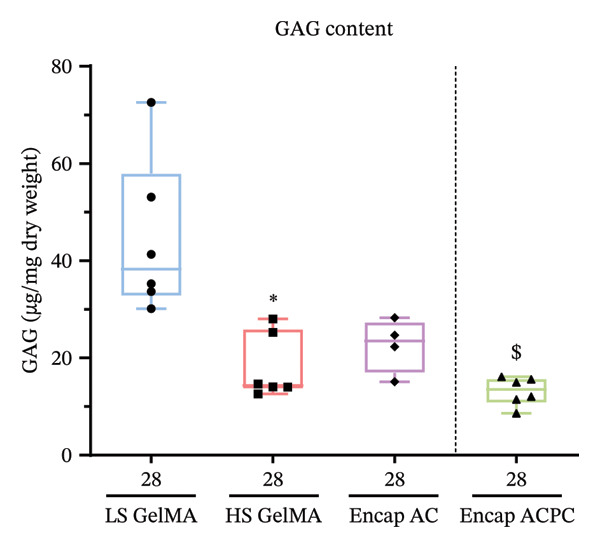
Encapsulation of cells in agarose microgels does result in less GAG production on the global scale. The GAG content in all experimental groups at Day 28 subtracted by the mean GAG content at Day 0. Between experimental groups, ^∗^ indicates a significant difference to LS GelMA, and $ indicates a significant difference to Encap AC at the same timepoint. The exact *p*‐values can be found in Supporting Table 2.

GAG production was observed in all groups (Figure 8). Chondrocytes embedded in LS GelMA showed significantly more mean GAG production than in HS GelMA (*p* = 0.0030, Kruskal–Wallis), which agrees with the hypothesis. Besides, encapsulation of chondrocytes in agarose microgels did not lead to increased GAG production, as HS GelMA has a similar GAG content to Encap AC, and the mean value was less than in LS GelMA, although not significantly lower (*p* = 0.1, Kruskal–Wallis). Thus, improvement of the PCM due to cell encapsulation did not directly result in increased GAG content.

### 3.5. Cell Encapsulation Improves ECM Synthesis in the Bottom Region

As depth‐dependent differences in cell numbers and PCM quality were observed earlier, the effect of depth was also determined for ECM production. The Type‐II collagen immunofluorescent staining (Figure 6A,C,E,G) and alcian blue staining (Figure 6B,D,E,H) revealed ECM production in all experimental groups. Visually, large differences in ECM synthesis were observed both between the regions and experimental groups. Overall, LS GelMA clearly produced the most alcian blue staining compared to the other groups. However, large variations in cell content were observed (Figure 4B). Therefore, the stained areas were normalized to the number of nuclei to get insight into production per individual cell, as was done for the PCM analysis.

The comparison of both alcian blue and Type‐II collagen between experimental groups revealed that there was no significant difference in the amount of ECM produced per cell (Figure 6I). This indicates that the differences in staining can be attributed to region‐specific differences in cell proliferation (Figure 4B). The area of Type‐II collagen staining per cell significantly decreased with depth for both the LS GelMA (top vs middle: *p* = 0.042; top vs bottom: *p* = 0.014) and HS GelMA (top vs middle: *p* = 0.0015; top vs bottom: *p* = 0.0003, Figure 6J). Such tissue heterogeneity was absent for HS GelMA with Encap ACs.

These results suggest that the synthesis of ECM is depth‐dependent in LS and HS GelMA and that ECM production is hampered in the bottom regions of these constructs. Although the amount of staining is strongly dependent on the cell density, the lack of Type‐II collagen staining in the bottom regions is clear from the microscopy images (Figure 6A,C). When encapsulated, this lack of ECM synthesis in the bottom regions is not observed (Figure 6E,G), suggesting that tissue regeneration is more homogeneous.

### 3.6. Matrix Synthesis by Encap ACPCs Is Similar to Encap ACs

Since the clinical use of ACs is limited by the loss of the chondrogenic phenotype during *in vitro* expansion, ACPCs were investigated as an alternative cell source. Overall, limited differences were detected between the Encap ACs versus ACPCs in HS GelMA, and no cell type outperformed the other.

The DNA assay showed a significantly higher DNA content for the ACPCs (92.0 ± 19.0 ng/mg sample dry weight) compared to the ACs (38.7 ± 12.8 ng/mg sample dry weight, *p* = 0.0010, independent *t*‐test) on Day 0 (Figure 4A). The ACPCs showed slightly higher LDH activity in the early stages of culture compared to Encap ACs, being significant on Days 6 and 10 (*p* = 0.16 and *p* = 0.006, independent *t*‐test, respectively). The LDH activity of ACPCs did decrease significantly during the culture (Figure 4C). During the last 2 weeks of the culture, the LDH activity was significantly higher on Day 17, Day 20, and Day 28 in Encap AC compared to Encap ACPC (*p* = 0.04, Mann–Whitney for Day 17; *p* = 0.0020, *p* = 0.0030, independent *t*‐test for Day 20 and Day 28, respectively) but still far from the maximum LDH release (positive control).

The cell content in the top, middle, and bottom regions determined based on the microscopy images showed that the ACPC content was not depth‐dependent, as was observed for the Encap ACs. Additionally, no significant differences were found between ACs and ACPCs (Figure 4B).

Histology (Figure 5) and semiquantitative analysis (Figure 7) of the microscopy data revealed that the Encap ACs and ACPCs produced very similar PCMs. No significant differences in Type‐VI collagen (Figure 7A) and perlecan (Figure 7B) synthesis were found between the two cell types, although there was more variation in perlecan synthesis between samples within the Encap ACPC group. The analysis of the PCM completeness showed trends similar to the Encap ACs, without significant differences between the cell types (Figure 7C). The PCM coverage was not significantly different between the cell types and the regions (Figure 7C). Interestingly, the PCM coverage data distribution did reveal that there were more ACPCs (72.9%) with 75%–100% PCM coverage in the top region compared to the ACs (61.3%, Figure 7D).

When analyzing the ECM synthesis, both at the macro‐ and microscales, no major differences between the Encap ACs and ACPCs were observed. However, on Day 28, the GAG content of the ACPCs was slightly, albeit significantly, lower compared to the ACs (*p* = 0.0080, independent *t*‐test). On the contrary, the analysis of the alcian blue staining showed no significant differences in sGAG production (Figure 6I).

The analysis of the Type‐II collagen staining revealed that the synthesis by Encap ACPCs was similar throughout the depth of the construct, as seen with ACs. However, when comparing the two cell types, the mean Type‐II collagen staining per cell is higher for the ACPCs (top: 14.4 μm^2^/nuclei ± 3.95; middle: 12.3 μm^2^/nuclei ± 7.60; bottom: 11.5 μm^2^/nuclei ± 9.19). Nevertheless, these values were only significantly higher in the top region (*p* = 0.042) and the standard deviation of the ACPC samples in the lower regions was larger (Figure 6J).

## 4. Discussion

This study investigated the effect of microenvironmental mechanical stiffnesses on local ECM and PCM synthesis by ACs, by lowering only the microenvironmental stiffness, independent of the total construct stiffness. Additionally, the potential of ACPCs as an alternative to ACs in this system was explored. The results show that the soft microgel encapsulation in a stiffer bulk hydrogel allows for chondrogenic matrix synthesis and results in more homogeneous PCM synthesis by ACs throughout the depth of the tissue, although the total ECM matrix production (evaluated by GAG production) was still lower than that of the soft bulk hydrogel. The comparison between Encap ACs and ACPCs showed a very similar response of the two cell types, which supports using ACPCs as a potential alternative to ACs.

Although encapsulation did not demonstrate an advantageous effect on the overall amount of ECM produced in comparison with the soft hydrogel, the analysis of the microscopy data prominently showed that the depth dependency of matrix production observed within both the soft and stiff GelMA was not present after cell encapsulation. Both the structure of the PCMs (Figure 7C–E) and the Type‐II collagen synthesis (Figure 6J) showed depth dependency within the LS and HS GelMA, but not in the Encap AC group (although the absolute amount between gels is not substantially different). It is hypothesized that this effect is due to the difference in properties between the hydrogels, as the mechanical stiffness of a poroelastic material affects the distribution of strain when quickly (w.r.t. relaxation time constant) compressed in a time‐dependent way [52, 53]. Low‐stiffness materials display an uneven distribution of strain, due to the time‐dependent influence of the fluid fraction on the material deformation, causing an uneven deformation and strain distribution of the material under dynamic loading conditions. This was consistent with the increased ECM production in the top region of the LS GelMA, compared to the bottom regions, indicating that the observed matrix production is possibly load‐induced. On the contrary, strains are expected to be more evenly distributed in the HS GelMA, due to the larger relative influence of the elastic modulus compared to the porous response. As a result, the maximum local strain near the surface is expected to be lower, but these same strains to be higher deeper in the hydrogel, which could explain the more homogeneous matrix synthesis in HS GelMA.

By combining a soft microenvironment with a stiff bulk material, it appears that the strain from loading penetrated deep into the bulk hydrogel and that the volumetric strain may still have been amplified by encapsulation in a soft microgel, as is apparent from the increased local ECM production in the bottom regions of the constructs. This is reflective of the phenomenon observed in native articular cartilage [2, 14, 16, 17]. It should be noted, though, that many other factors, e.g., fluid flow and tensile strain, will change with the properties of the materials and that these also affect matrix synthesis. Additionally, the used bioreactor system was displacement‐controlled, causing variations in strain rate dependent on the thickness of the tissue. From the results of this study, the exact mechanical stimulation of the cells cannot be pinpointed. Further analysis of the bulk and local mechanics, using, e.g., finite element analysis or *in situ* imaging, is needed to associate matrix synthesis with changes in microenvironmental mechanical stimulation. Nevertheless, the results of this study suggest that the concept of combining a soft microgel with a stiff bulk hydrogel may have a favorable effect on matrix production throughout the tissue depth and potentially the quality of tissue‐engineered articular cartilage.

Besides the changes in the distribution of mechanical stimuli, GelMA and agarose interact differently with the cell. While agarose is nonadhesive, GelMA contains integrin‐binding domains like arginine–glycine–aspartic acid (RGD) sequences, which allow for cell adhesion [54]. A lack of cell adhesion to agarose could diminish the effect of mechanical stimulation, as the cell is unable to sense its environment. However, it is not expected that the lack of cell binding to agarose had major effects since the formed PCM provided cell adhesion sites before dynamic culture (Figure S4, Supporting Information, [30]). A lack of cell‐binding sites could even be favorable, since a more elongated cell morphology, associated with a loss of the chondrogenic phenotype, can be promoted by cell binding to low‐stiffness hydrogels [55]. The materials used in this study were therefore considered to be sufficient to promote the chondrogenic phenotype.

However, the mechanism chosen to create the difference in the hydrogel stiffness potentially causes other differences, the most important of which is the diffusion of matrix proteins. To increase the material stiffness, the crosslinking density of the GelMA was increased. This can decrease the porosity of the hydrogel and affect the diffusion of newly synthesized matrix [56]. In HS GelMA, smaller regions of especially the ECM were observed compared to LS GelMA, which is in line with observations in literature [27, 29, 57]. In this context, it is likely that the agarose used has a smaller pore size compared to GelMA [56, 58], leading to matrix accumulation in the microgels and possibly limiting diffusion from the agarose to GelMA [59, 60]. This could explain the limited ECM staining observed in the encapsulated groups. Altogether, in the future, the properties of the bulk and microgel materials should be adjusted to facilitate optimal formation and diffusion of PCM and ECM, which is possible in the exploitation of the presented system.

Our study demonstrated that the perlecan staining and PCM coverage after cell encapsulation was improved. The combined presence of Type‐VI collagen and perlecan is associated with the mechanical properties of the PCM [14, 61]. Additionally, perlecan is essential for modulating the activity of growth factors and cell–matrix interactions [62, 63]. As the presence of perlecan increased after encapsulation, it may be assumed that the higher perlecan presence results in more native‐like functioning of the PCM. Additionally, the improved PCM coverage around the ACs after encapsulation is considered essential to the functionality of the PCMs, since partial coverage by the PCM can diminish its mechanical functionality [64]. Together, the results suggest that encapsulation allowed better PCM maturation within 28 days. Although there is literature suggesting that perlecan synthesis is regulated by changes in substrate stiffness and mechanical stimulation [64, 65] and is independent of TGF‐β stimulation [66], the exact regulatory systems of perlecan during chondrogenesis are largely unknown [24]. Investigating the underlying regulatory mechanisms of perlecan synthesis and PCM structuring would allow for the fine‐tuning of microenvironmental material properties to stimulate optimal PCM synthesis.

It should be noted that there were some limitations to the histological stainings used in this study. Ideally, the interface of the hydrogel with the surrounding tissue would have been presented. However, when preparing the samples for histology, the hydrogels shrank and detached from the surrounding tissue. Therefore, the surrounding tissue was not shown in this manuscript. The structural quantification of the PCM was based on the staining outside of the cell nucleus, as was done in our previous study [30]. Ideally, a cell membrane staining would have been used for these quantifications. In this study, the same Type‐VI collagen antibody was used as before, where it was shown to specifically stain extracellular collagen in the PCM. However, we did not use confocal microscopy (due to time limitations and microscope availability), and thus, parts of the PCM within the section were projected over the intracellular area. This was confirmed by taking a confocal image of an Encap AC sample (Supporting Figure 5S) where the extracellular nature of the Type VI collagen and perlecan labeling are demonstrated.

Considering the potential advantageous effects on both ECM and PCM synthesis after cell encapsulation while maintaining bulk stiffness, the presented system opens up interesting opportunities for articular cartilage tissue engineering. Since the cells are encapsulated, there is no contact with the bulk material. This creates great flexibility in choosing the bulk material properties most suitable for the distribution of mechanical stimuli and the diffusion of matrix proteins. This enables a wider use of synthetic biomaterials, which have tunable mechanical properties but currently have biocompatibility problems [67, 68]. More importantly, the microenvironment can be tuned for optimal stimulation of the cells, both mechanically and chemically. Local integration of growth factors, allowing their local (slow) release, could make preculture, as done in the current study, obsolete [69]. Cells requiring growth factor stimulation for chondrogenic differentiation, like ACPCs, can be stimulated *in situ*, shortening culture times and decreasing treatment costs.

The apparent modulus of the LS GelMA on Day 0 was higher than the elastic modulus measured when the hydrogel was characterized. It is hypothesized that this difference is due to the handling of the hydrogel and the subsequent cooling of the hydrogel, increasing the stiffness of the material. Based on the pre‐experimental characterization, we aimed for a one magnitude difference in material stiffness between LS and HS GelMA, but only a three times difference was achieved. Nevertheless, the biological response was still apparent and no differences in conclusions are anticipated.

When aiming to improve articular cartilage tissue engineering strategies, the cell source is an important factor to consider. Since the amount of autologous ACs that can be collected is limited, *in vitro* expansion is necessary, causing the loss of the chondrogenic phenotype at high passage numbers [8, 32]. ACPCs are a potential alternative cell source as they can be easily harvested from the surface of the tissue and the chondrogenic phenotype is retained at high passage numbers [33, 35]. In this study, no major differences in PCM and ECM synthesis were found between Encap ACs (P1) and ACPCs (P3), although a lower total GAG content was measured for the ACPCs after 28 days. Culture of ACPCs in the LS and HS GelMA conditions could be valuable in future studies to get more insight into the behavior of these cells in differed 3D substrate stiffnesses. To note, the current population of ACPCs shows large heterogeneity in performance (synthesis of ECM and PCM) between individual cells [30, 34]. Using a subpopulation of ACPCs with distinct properties that are passaged before exploitation presents a strategy that may contribute to improved outcomes. Additional analyses to quantify metalloproteinase (MMP) activity and cytokine production by ACs and ACPCs could provide more understanding into the behavior of these cells and reveal possible drawbacks of either cell source. Taken together, the results of this study suggest that ACPCs are a potential alternative to ACs in the current system but require further optimization for their use.

Another limitation of our study is the longer preculture time of the encapsulation groups. To allow chondrogenic stimulation of the ACPCs, a preculture with BMP‐9 was needed [37]. To keep the culture times of the Encap ACs the same, these cells were precultured for a similar time with TGF‐β and dexamethasone stimulation. Additionally, all samples were allowed to rest for 2 days before mechanical loading was started. From our previous study, we know that ACs and ACPCs form a well‐structured PCM within 5 days in agarose microgels [30]. The presence of a PCM when mechanical stimulation is started can have a significant effect on chondrogenic matrix synthesis and the expression of cytokines and MMPs [64]. Therefore, it should be considered that the encapsulated cells had a more advantageous environment at the start of the culture.

Taken together, the results of this study suggest that the presented method of cell encapsulation has unique exploitable aspects for articular cartilage tissue engineering. Combining a stiff bulk hydrogel with soft cell‐laden microgels allowed local PCM maturation and more homogenous tissue production, even in the deeper regions of the construct although this was still in total less than in the soft bulk hydrogel. If development is continued, the system could be optimized to create the ideal microenvironment for cells to thrive and accelerate neocartilage formation. While this study mainly focused on using native‐like PCM mechanical properties of the microgels, the local chemical composition, structure, porosity, and local release of growth factors and/or drugs can all have a great influence on cell behavior and matrix synthesis. Fine‐tuning of the cell microenvironment would allow for meeting specific requirements based on cell type and tissue depth. This can create opportunities for targeted cell stimulation and creating articular cartilage tissue‐engineered constructs, which are functional upon implantation and allow for durable tissue regeneration.

Overall, while the softer bulk hydrogel stimulated superior ECM production, its distribution with depth was less homogenous. In this context, the presented method of cell encapsulation in combination with the ability to differentially tune bulk hydrogel properties shows potential by altering mechanical stimulation through the bulk hydrogel while still providing another local condition for the cells. However, further investigation is necessary to fully evaluate its capacity and the potential to optimize cartilage tissue engineering outcomes.

## Author Contributions

Marloes van Mourik: conceptualization, methodology, investigation, formal analysis, validation, visualization, writing–original draft, and writing–review and editing.

Janne Spierings: conceptualization, methodology, investigation, formal analysis, validation, visualization, writing–original draft, and writing–review and editing.

Pinar Koca: resources, methodology, investigation, and writing–review and editing.

Gabriele Addario: investigation and visualization.

Florencia Abinzano: writing–review and editing.

Corrinus C. van Donkelaar: writing–review and editing.

Keita Ito: conceptualization, funding acquisition, supervision, and writing–review and editing.

Jasper Foolen: conceptualization, funding acquisition, supervision, and writing–review and editing.

## Funding

This work was financially supported by the Gravitation Program “Materials Driven Regeneration” and funded by the Netherlands Organization for Scientific Research (024.003.013) and the European Union’s Horizon 2020 Research and Innovation Programme (Grant No. 952981).

## Conflicts of Interest

The authors declare no conflicts of interest.

## Supporting Information

Additional supporting information can be found online in the Supporting Information section.

## Supporting information


**Supporting Information** The supporting information file provides additional data depicting the regions of interest used for the microscopy image analysis (Figure S1), the apparent modulus at day 0 (Figure S2), the stainings of PCM (Type‐VI collagen, perlecan, Figure S3), ECM components of samples on Day 0, and the *p*‐values of all significant differences found (Type‐II collagen, sGAGs, Figure S4), and representative confocal imaging of PCM staining (Type‐VI collagen and perlecan, Figure 5S).

## Data Availability

All data used to support the findings of this study are available from the corresponding author upon request.
