# Supplementary material for: Mechanical Tuning of the Cell Microenvironment Using a Biomimetic Hydrogel System for Articular Cartilage Tissue Engineering
Source: J Tissue Eng Regen Med. 2026 May 31;2026:9947868. doi: 10.1155/term/9947868 (PMC13239536; doi:10.1155/term/9947868)
Supplement: Supplementary file 1 — Supporting Information The supporting information file provides additional data depicting the regions of interest used for the microscopy image analysis (Figure S1), the apparent modulus at day 0 (Figure S2), the stainings of PCM (Type‐VI collagen, perlecan, Figure S3), ECM components of samples on Day 0, and the p‐values of all significant differences found (Type‐II collagen, sGAGs, Figure S4), and representative confocal imaging of PCM staining (Type‐VI collagen and perlecan, Figure 5S). [file TERM-2026-9947868-s001.zip › R4_Supplementary material_final.docx]

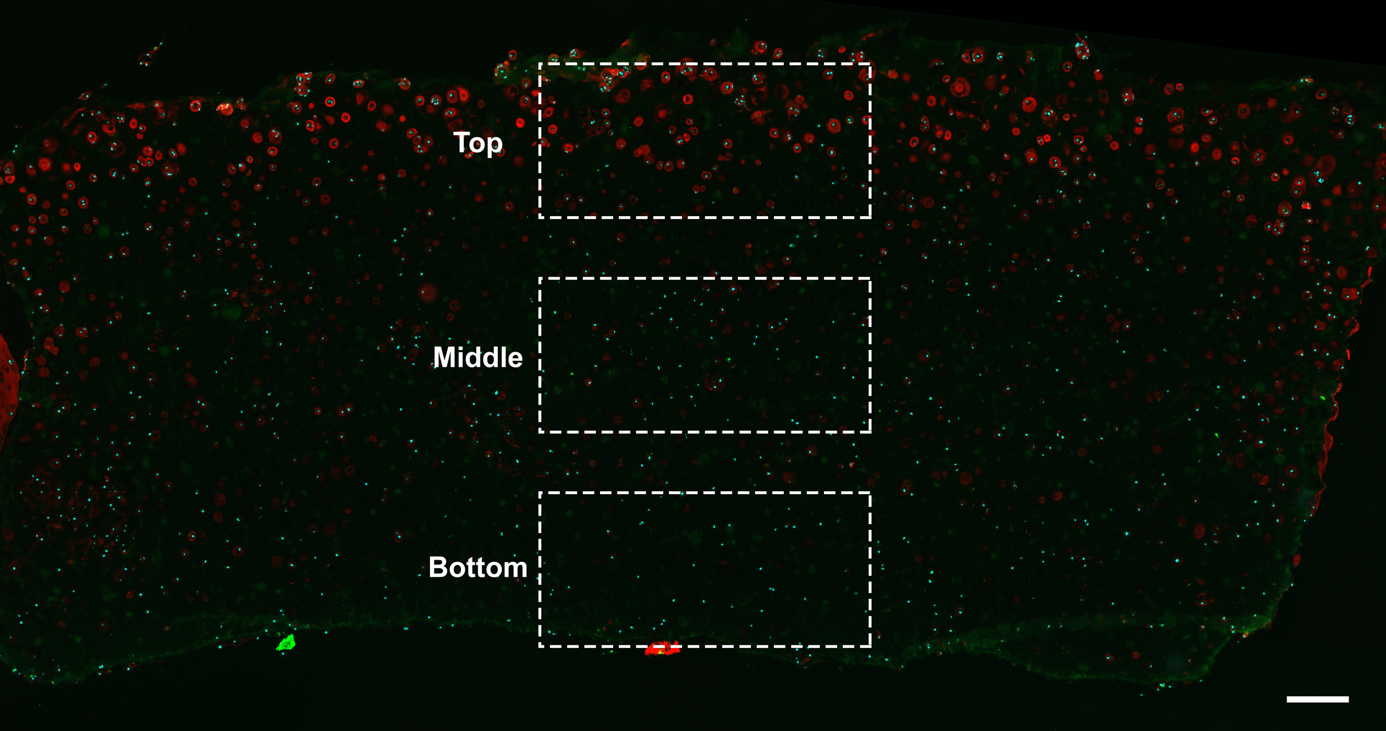


**Fig. S1** Regions of interest at the top, middle, and bottom of the hydrogel constructs selected for the microscopy image analysis. Scale bar = 200 µm


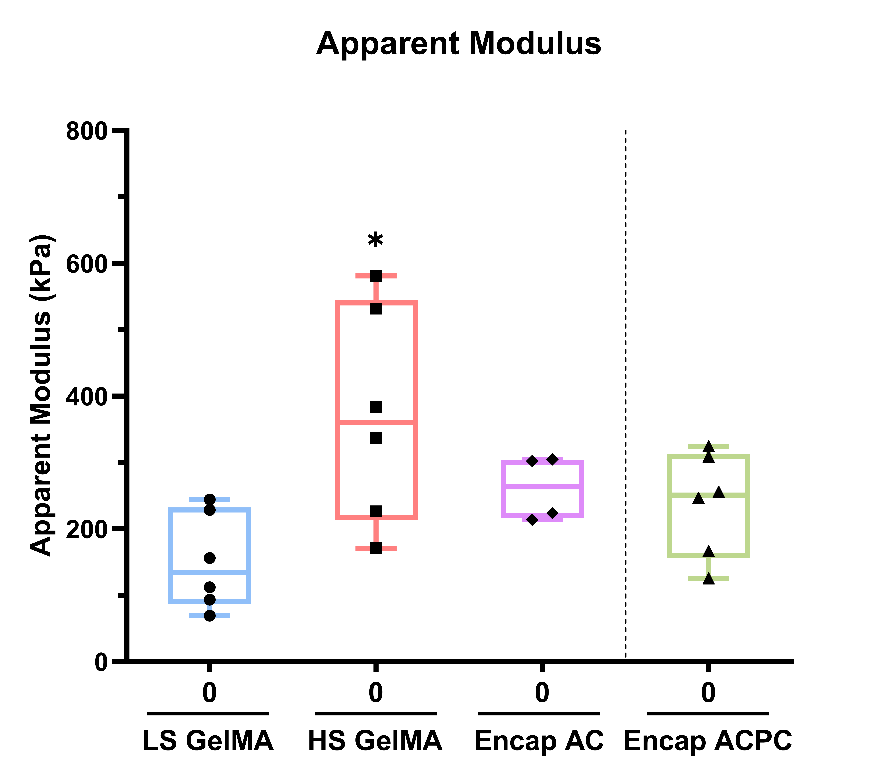


**Fig. S2** The apparent modulus of all experimental groups at day 0 calculated from indention tests using the Hertz contact model. This graph shows cycle 3, as a representation of all 5 cycles. Between experimental groups, * indicates a significant difference to LS GelMA.


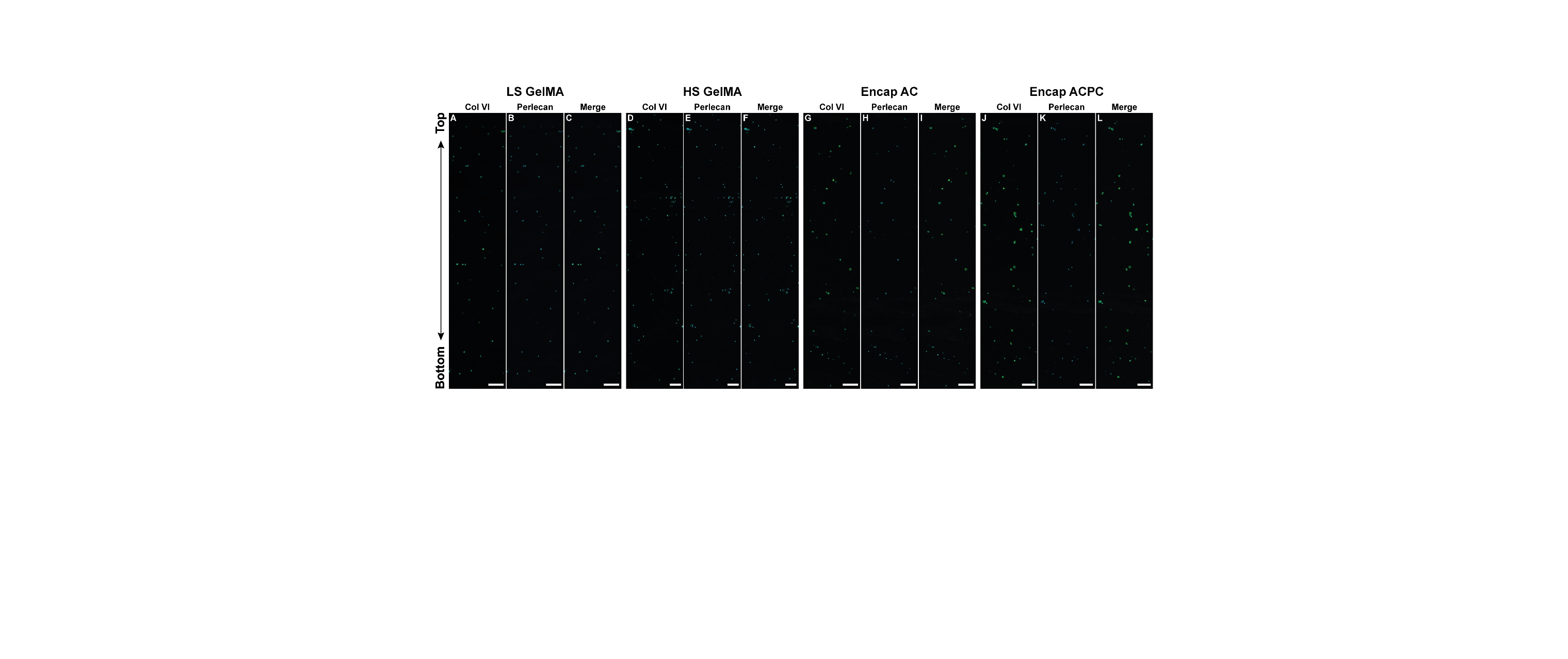


**Fig. S3** The staining of PCM components on day 0 showed minimal type-VI collagen in LS and HS GelMA and more developed PCMs in the encapsulated groups. Immunofluorescent stainings of type-VI collagen (green; A, D, G, H), perlecan (magenta; B, E, H, K), and a combined projection (C, F, I, L) with cell nuclei (cyan) are depicted. Scale bar = 100 µm.


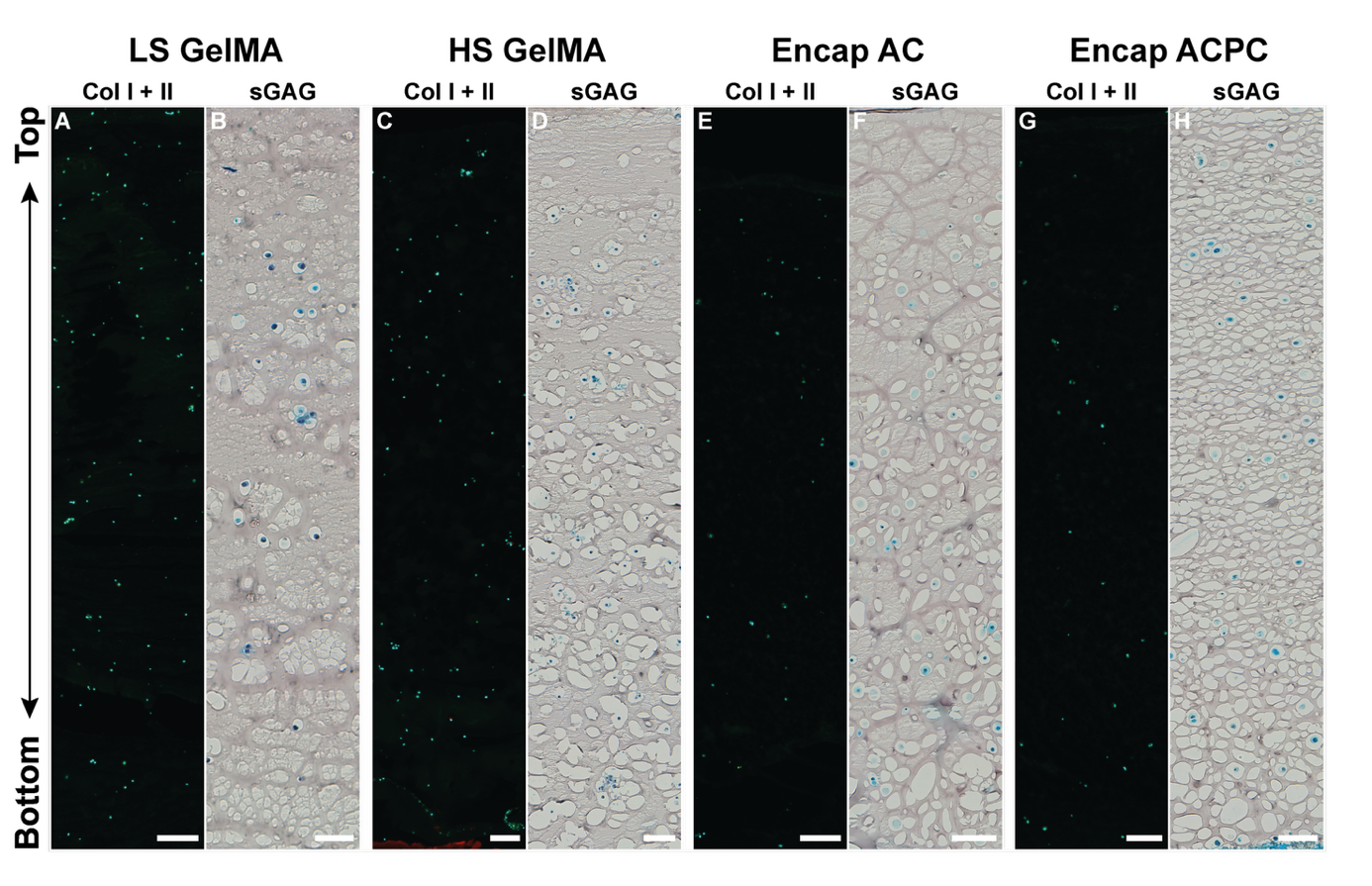


**Fig. S4** Staining of ECM components showed the absence of type-II collagen and sGAGs on day 0. Agarose microgels are visible with the alcian blue staining (F, H). Immunofluorescent stainings of type-I collagen (gray), type-II collagen (red**),** and cell nuclei (cyan) are depicted in A, C, E, and G. Alcian blue staining for sGAGs is depicted in B, D, F, and H. Scale bar = 100 µm.

**Fig. S5** A representative condition Encap AC was stained and imaged using the SP8X confocal microscope, with a 63x objective (Leica Microsystems). Immunofluorescent stainings of type-VI collagen (green; A) and perlecan (magenta; B) were first performed, together with a combined staining (C) with cell nuclei (cyan). Scale bar = 50 µm.

**Supplementary Table 1.** *p*-values of all significant differences within the experimental groups.

|  |  | **LS GelMA** | **HS GelMA** | **Encap AC** | **Encap ACPC** |
| --- | --- | --- | --- | --- | --- |
| **DNA content** | **Day 0 vs. Day 28** |  |  | *p* = 0.014 (t-test) |  |
| **Cell content** | **Top vs. Middle** | *p* = 0.018 (RM ANOVA) |  |  |  |
|  | **Top vs. Bottom** | *p* = 0.005 (RM ANOVA) |  | *p* = 0.012 (Friedman) |  |
| **Cell death** | **Day 3 vs.**  **Day 17** |  |  |  | *p* = 0.012 (Friedman) |
|  | **Day 3 vs. Day 24** |  | *p* = 0.041 (Friedman) |  |  |
|  | **Day 6 vs. Day 17** |  |  |  | *p* = 0.0006 (Friedman) |
|  | **Day 6 vs. Day 20** |  |  |  | *p* = 0.005 (Friedman) |
|  | **Day 6 vs. Day 28** |  |  |  | *p* = 0.005 (Friedman) |
|  | **Day 10 vs.**  **Day 17** |  |  |  | *p* = 0.027 (Friedman) |
|  | **Day 17 vs.**  **Day 28** |  |  | *p* = 0.050 (Mixed effect) |  |
| **Type-VI Collagen** | **Top vs. Middle** |  | *p* = 0.038 (RM ANOVA) |  |  |
| **PCM Thickness** | **Top vs. Middle** | *p* = 0.005 (RM ANOVA) | *p* = 0.049 (RM ANOVA) |  | *p* = 0.026 (RM ANOVA) |
|  | **Top vs. Bottom** | *p* = 0.0005  (RM ANOVA) | *p* = 0.0007 (RM ANOVA) |  |  |
|  | **Middle vs. Bottom** |  | *p* = 0.025 (RM ANOVA) |  |  |
| **PCM Coverage** | **Top vs. Middle** | *p* = 0.008 (RM ANOVA) |  |  |  |
|  | **Top vs. Bottom** | *p* = 0.005 (RM ANOVA) | *p* = 0.0007 (RM ANOVA) |  |  |
|  | **Middle vs. Bottom** |  | *p* = 0.008 (RM ANOVA) |  |  |
| **Type-II Collagen** | **Top vs. Middle** | *p* = 0.042 (RM ANOVA) | *p* = 0.002  (RM ANOVA) |  |  |
|  | **Top vs. Bottom** | *p* = 0.014 (RM ANOVA) | *p* = 0.0003 (RM ANOVA) |  |  |

**Supplementary Table 2.** *p*-values of all significant differences between the experimental groups.

|  |  | **LS GelMA vs.  HS GelMA** | **LS GelMA vs. Encap AC** | **HS GelMA vs. Encap AC** | **Encap AC vs.  Encap ACPC** |
| --- | --- | --- | --- | --- | --- |
| **Apparent Modulus** | **Day 0** | *p* = 0.01  (one-way ANOVA) |  |  |  |
| **DNA content** | **Day 0** | *p* = 0.03  (one-way ANOVA) |  | *p* = 0.002  (one-way ANOVA) | *p* = 0.001  (t-test) |
|  | **Day 28** |  | *p* = 0.003  (one-way ANOVA) | *p* = 0.013  (one-way ANOVA) | *p* = 0.019  (Mann-Whitney) |
| **Cell content** | **Top** | *p* = 0.003  (one-way ANOVA) | *p* = 0.046  (one-way ANOVA) |  |  |
|  | **Middle** |  | *p* = 0.002  (one-way ANOVA) |  |  |
|  | **Bottom** |  | *p* = 0.013  (one-way ANOVA) |  |  |
| **Cell death** | **Day 6** |  |  |  | *p* = 0.016  (t-test) |
|  | **Day 10** |  |  |  | *p* = 0.006  (t-test) |
|  | **Day 13** |  | *p* = 0.005  (one-way ANOVA) | *p* = 0.008  (one-way ANOVA) |  |
|  | **Day 17** |  | *p* = 0.007  (Kruskal-Wallis) |  | *p* = 0.038  (Mann-Whitney) |
|  | **Day 20** |  | *p* = 0.028  (Kruskal-Wallis) | *p* = 0.028  (Kruskal-Wallis) | *p* = 0.002  (t-test) |
|  | **Day 24** |  |  | *p* = 0.006  (Kruskal-Wallis) |  |
|  | **Day 28** |  | *p* < 0.0001  (one-way ANOVA) | *p* < 0.0001  (one-way ANOVA) | *p* = 0.0003  (t-test) |
| **Type-VI Collagen** | **Top** |  | *p* = 0.017  (one-way ANOVA) |  |  |
| **Perlecan** | **Top** |  | *p* = 0.003  (one-way ANOVA) | *p* = 0.015  (one-way ANOVA) |  |
|  | **Middle** |  | *p* = 0.020  (Kruskal-Wallis) | *p* = 0.205  (Kruskal-Wallis) |  |
|  | **Bottom** |  | *p* = 0.008  (one-way ANOVA) | *p* = 0.006  (one-way ANOVA) |  |
| **PCM Thickness** | **Top** |  | *p* = 0.028  (one-way ANOVA) | *p* = 0.002  (one-way ANOVA) |  |
|  | **Bottom** |  | *p* = 0.033  (Kruskal-Wallis) | *p* = 0.004  (Kruskal-Wallis) |  |
| **PCM Coverage** | **Middle** |  | *p* = 0.007  (one-way ANOVA) |  |  |
|  | **Bottom** |  | *p* = 0.015  (Kruskal-Wallis) | *p* = 0.003  (Kruskal-Wallis) |  |
| **Type-II Collagen** | **Top** |  |  |  | *p* = 0.042  (t-test) |
