# Supplementary figures and images for: Mechanical Tuning of the Cell Microenvironment Using a Biomimetic Hydrogel System for Articular Cartilage Tissue Engineering
Source: J Tissue Eng Regen Med. 2026 May 31;2026:9947868. doi: 10.1155/term/9947868 (PMC13239536; doi:10.1155/term/9947868)

# Apparent Modulus

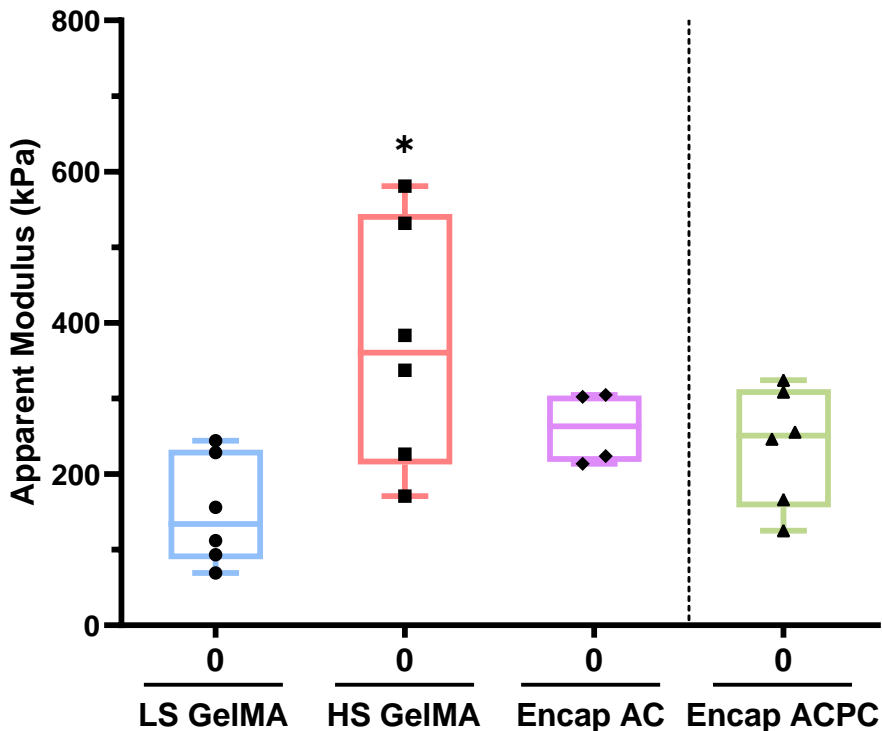

Supplement: Supplementary file 1 — Supporting Information The supporting information file provides additional data depicting the regions of interest used for the microscopy image analysis (Figure S1), the apparent modulus at day 0 (Figure S2), the stainings of PCM (Type‐VI collagen, perlecan, Figure S3), ECM components of samples on Day 0, and the p‐values of all significant differences found (Type‐II collagen, sGAGs, Figure S4), and representative confocal imaging of PCM staining (Type‐VI collagen and perlecan, Figure 5S). [file TERM-2026-9947868-s001.zip › Fig S2.pdf]

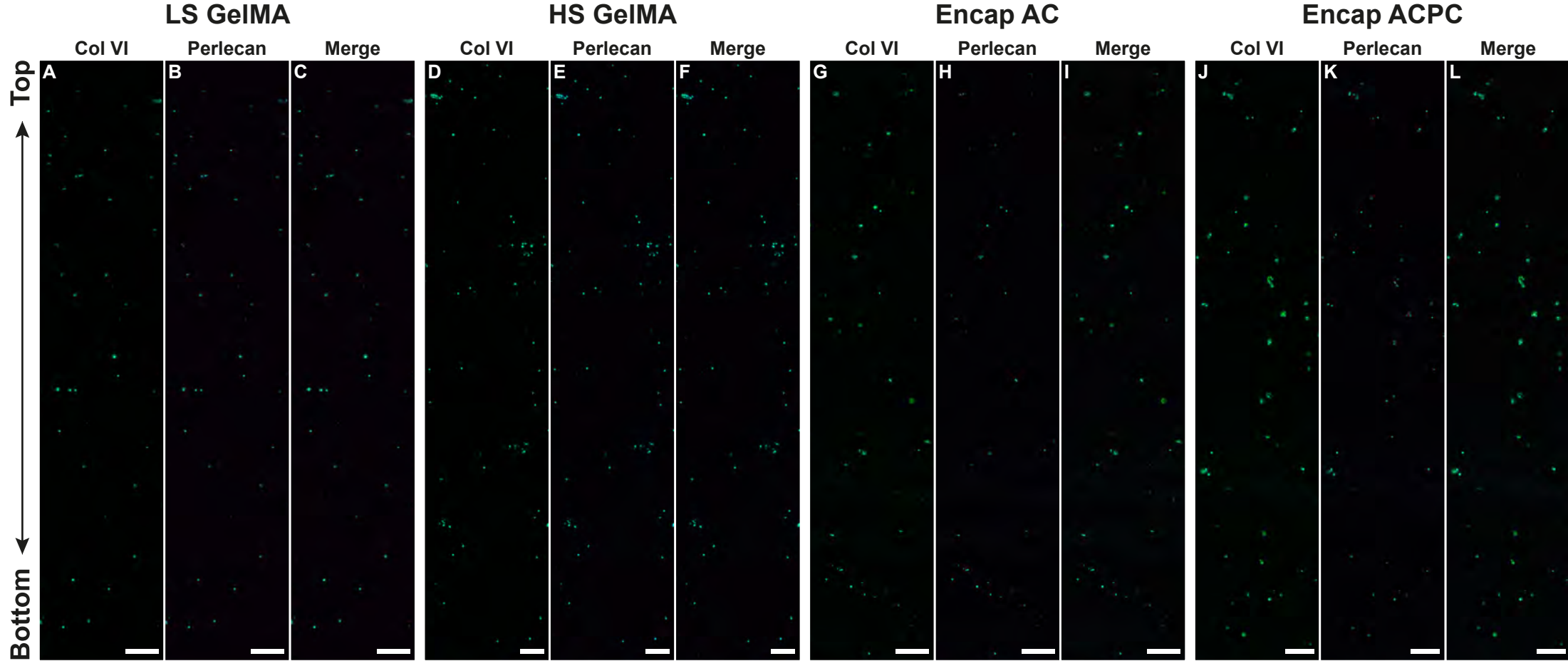

Supplement: Supplementary file 1 — Supporting Information The supporting information file provides additional data depicting the regions of interest used for the microscopy image analysis (Figure S1), the apparent modulus at day 0 (Figure S2), the stainings of PCM (Type‐VI collagen, perlecan, Figure S3), ECM components of samples on Day 0, and the p‐values of all significant differences found (Type‐II collagen, sGAGs, Figure S4), and representative confocal imaging of PCM staining (Type‐VI collagen and perlecan, Figure 5S). [file TERM-2026-9947868-s001.zip › Fig S3_sm.pdf]

## LS GelMA

## HS GelMA

## Encap AC

## Encap ACPC

Col I + II

sGAG

Top

Bottom

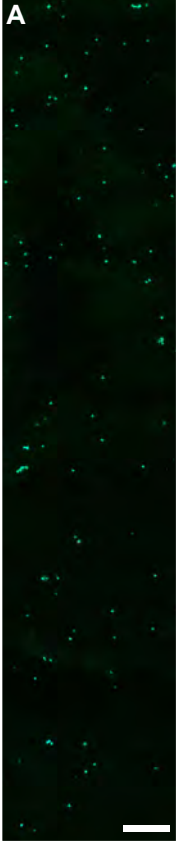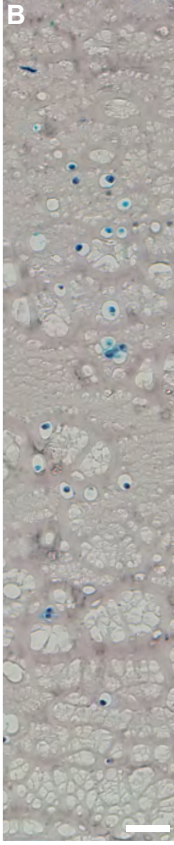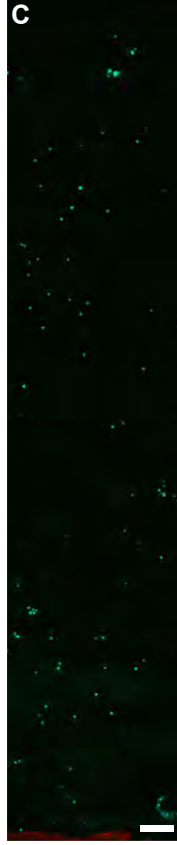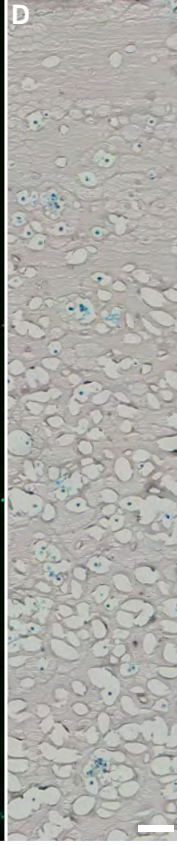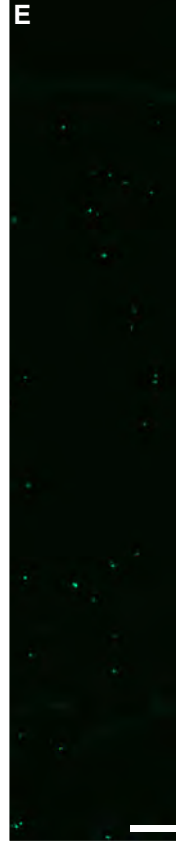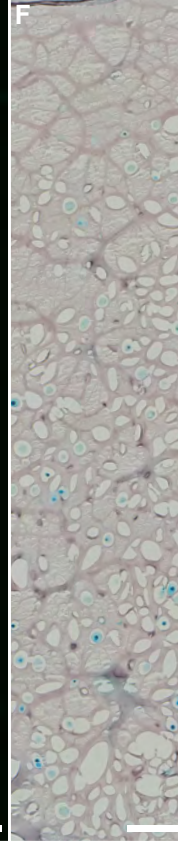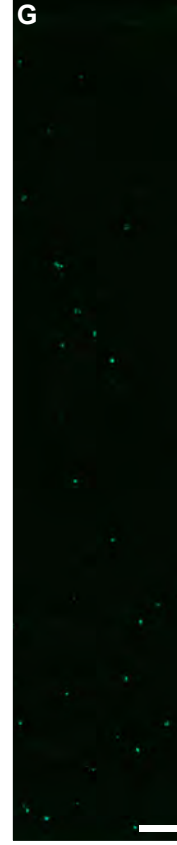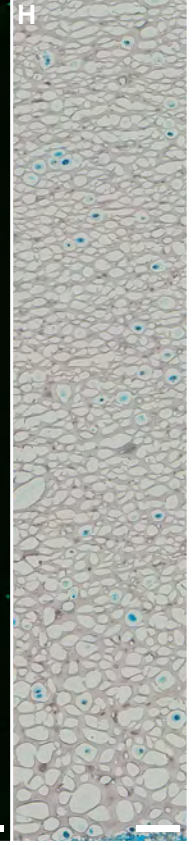

Supplement: Supplementary file 1 — Supporting Information The supporting information file provides additional data depicting the regions of interest used for the microscopy image analysis (Figure S1), the apparent modulus at day 0 (Figure S2), the stainings of PCM (Type‐VI collagen, perlecan, Figure S3), ECM components of samples on Day 0, and the p‐values of all significant differences found (Type‐II collagen, sGAGs, Figure S4), and representative confocal imaging of PCM staining (Type‐VI collagen and perlecan, Figure 5S). [file TERM-2026-9947868-s001.zip › Fig S4_sm.pdf]
