# Supplementary material for: Mechanical Tuning of the Cell Microenvironment Using a Biomimetic Hydrogel System for Articular Cartilage Tissue Engineering
Source: J Tissue Eng Regen Med. 2026 May 31;2026:9947868. doi: 10.1155/term/9947868 (PMC13239536; doi:10.1155/term/9947868)
Supplement: Supplementary file 1 — Supporting Information The supporting information file provides additional data depicting the regions of interest used for the microscopy image analysis (Figure S1), the apparent modulus at day 0 (Figure S2), the stainings of PCM (Type‐VI collagen, perlecan, Figure S3), ECM components of samples on Day 0, and the p‐values of all significant differences found (Type‐II collagen, sGAGs, Figure S4), and representative confocal imaging of PCM staining (Type‐VI collagen and perlecan, Figure 5S). [file Fig S1.pdf › Fig S1.pdf]

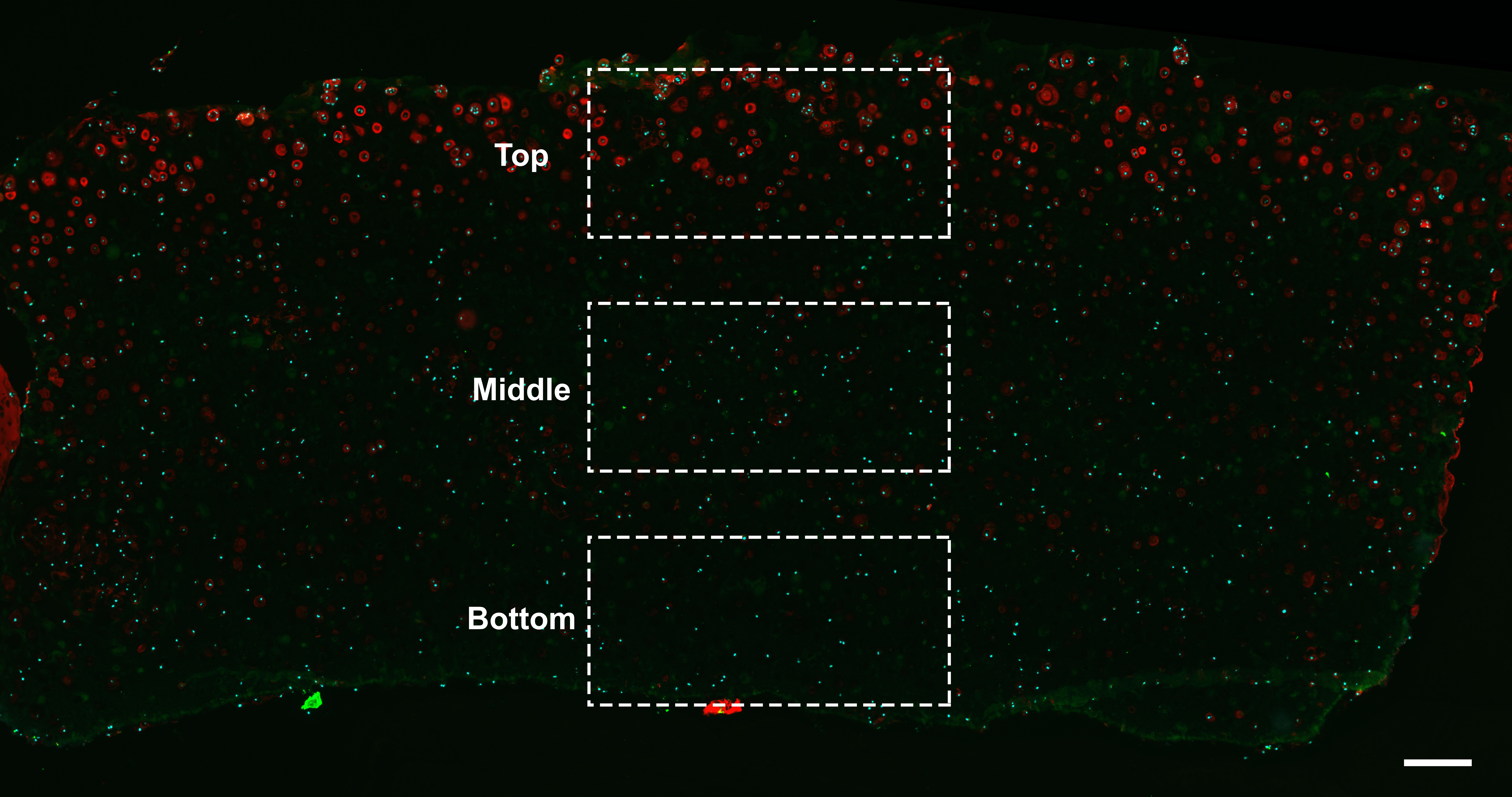
Top
Middle
Bottom
